# Basing care reforms on evidence: The Kenya health sector costing model

**DOI:** 10.1186/1472-6963-11-128

**Published:** 2011-05-27

**Authors:** Steffen Flessa, Michael Moeller, Tim Ensor, Klaus Hornetz

**Affiliations:** 1 University of Greifswald, Faculty of Law and Economics, Friedrich-Loeffler-Str. 70, D-17489 Greifswald, Germany; 2 German International Cooperation (GIZ), Health Sector Programme, Nairobi, Kenya; 3 Oxford Policy Management (OPM), Oxford, UK

## Abstract

**Background:**

The Government of the Republic of Kenya is in the process of implementing health care reforms. However, poor knowledge about costs of health care services is perceived as a major obstacle towards evidence-based, effective and efficient health care reforms. Against this background, the Ministry of Health of Kenya in cooperation with its development partners conducted a comprehensive costing exercise and subsequently developed the Kenya Health Sector Costing Model in order to fill this data gap.

**Methods:**

Based on standard methodology of costing of health care services in developing countries, standard questionnaires and analyses were employed in 207 health care facilities representing different trustees (e.g. Government, Faith Based/Nongovernmental, private-for-profit organisations), levels of care and regions (urban, rural). In addition, a total of 1369 patients were randomly selected and asked about their demand-sided costs. A standard step-down costing methodology was applied to calculate the costs per service unit and per diagnosis of the financial year 2006/2007.

**Results:**

The total costs of essential health care services in Kenya were calculated as 690 million Euros or 18.65 Euro per capita. 54% were incurred by public sector facilities, 17% by Faith Based and other Nongovernmental facilities and 23% in the private sector. Some 6% of the total cost is due to the overall administration provided directly by the Ministry and its decentralised organs. Around 37% of this cost is absorbed by salaries and 22% by drugs and medical supplies. Generally, costs of lower levels of care are lower than of higher levels, but health centres are an exemption. They have higher costs per service unit than district hospitals.

**Conclusions:**

The results of this study signify that the costs of health care services are quite high compared with the Kenyan domestic product, but a major share are fixed costs so that an increasing coverage does not necessarily increase the health care costs proportionally. Instead, productivity will rise in particular in under-utilized private health care institutions. The results of this study also show that private-for-profit health care facilities are not only the luxurious providers catering exclusively for the rich but also play an important role in the service provision for the poorer population. The study findings also demonstrated a high degree of cost variability across private providers, suggesting differences in quality and efficiencies.

## Background

In the first three decades after independence the people of the Republic of Kenya enjoyed an impressive improvement in all health indicators. For instance, from 1960 to 1990 life expectancy rose from 43 to 62 years, infant mortality dropped during this period from 122 to 63 and under-five-mortality declined from 204 to 93 per 1000 live births [1]. However, most indicators showed a deterioration during the 1990s, so that by the year 2000 the life expectancy was back to 49 years, infant mortality was some 83 and under-five-mortality some 134 per 1000 live births [2]. Main reasons for this deterioration are the AIDS epidemic [3], the manifestation of resistant malaria [4], the epidemiological transition with an increasing burden of chronic-degenerative diseases [5]. Strong inequity between the poor and the rich and rapid population growth are underlying factors driving negative health trends [6]. Whereas Kenyan health budgets had risen in absolute terms until 2005 [7], the health care services for the majority of rural and urban poor has deteriorated.

The Government of the Republic of Kenya realized this negative development and responded with health care reform. Based on a comprehensive "Health Policy Framework" (1994) two "National Health Sector Strategic Plans" (HSSP I: 1999-2004; HSSP II: 2009-2010) were approved, the latter building the cornerstone of the Kenyan health care reform. HSSP II provided the blueprint to innovations like the definition of a "Kenya Essential Package of Health" (KEPH), a "Community Strategy", a "Joint Framework of Work and Financing" (JPWD) as expression of the Sector-Wide Approach (SWAp) and a "Hospital Reform" aiming at more autonomy for provincial hospitals. The process of adapting the health care system is seen in perspectives of the so-called "Vision 2030", stating affordability, equity, quality and capacity as the main objectives of the entire social sector. Implementing the new constitution which was promulgated in August 2010 will equally impact on the system design of the Kenyan Health Sector, especially in terms of decentralisation and realising the 'Right to Health' [8].

Meanwhile the official health indicators have improved, e.g., life expectancy is 52 years, infant mortality rate is 52 and under-five-mortality rate is 77 per 1000 live births [9]. A part of these improvements can, however, be attributed to widescale, well-financed, vertical, disease-specific programmes (e.g. to combat malaria). Another part can is - at least partly - a consequence of the health care reform, such as the improvement of the supply chain management of essential drugs, the definition and application of an essential health care package, the reduction of user fees, the training of hospital managers, the direct allocation and distribution of funds etc. - a process that is under the leadership of the Ministries of Health and that is strongly supported by international development partners. The Ministries and the related partners have agreed on that all elements of health care reforms should be evidence-based, i.e., there is a strong need for sound epidemiological and economic data.

During the last few years the knowledge of demographic and disease-related statistics has improved due to strong investments into the Health Management Information System of Kenya [10]. However, the knowledge of the costs of health care services in this country is very limited, and this „scarcity of information inhibits governments from making informed choices about the allocation of public resources for better health, as well as improvements in the management of publicly provided and/or financed services" [11]. Consequently, the following questions could not be answered until the here presented costing model was developed:

- How high are the actual costs of health care services in total?

- How high are the actual costs of health care services on each level of service?

- Which role do the different trustees (Government, Private-for-Profit, Non-Profit) play?

- What are the unit costs for treating specific diseases on each level, e.g. what resources do we have to invest to treat one malaria case in a dispensary, a health centre and a district hospital?

- How much would it cost to provide the Kenya Essential Package of Health to every Kenyan, i.e., how would the costs react on an increasing demand?

It is obvious that decision-makers on health care reforms should know the answers to these questions in order to provide sufficient funds and allocate them to the most efficient levels of care. However, this information as not available in Kenya as the cost accounting of health care institutions is rudimentary and no national statistics exist on the micro-level distinguishing diseases and levels of care. Consequently, the National Health Accounts cannot provide an answer to these essential questions. It became necessary to develop a costing tool for Kenya and inform the policy process and in particular the health financing reforms with sufficiently precise costing information.

This paper presents the basic structure of the costing model and some basic results. In the next section we present a literature review of costing studies for health care services in developing countries. It is demonstrated that our knowledge of costs is very limited and is usually based on extremely small samples. The third section describes the Kenyan Health Sector Costing Model. The basic costing methodology is discussed as well as the process of data collection and analysis. The fourth section gives an overview of the basic findings and provides answers to the questions raised above. In the fifth section we discuss these findings and give some ideas how they can be utilized to inform the policy process. We conclude with some recommendations for the future development of costing health care services in Kenya.

## State-of-the-Art

In countries where accounting information is complete and reliable, costing of health care services is a standard. For instance, Mogyorosy & Smith [12] prepared a detailed literature review of methodological issues in costing health care services in EU states. They conclude that differences in costing methodologies are due to different decision situations and research questions, not due to disagreement on methodology and concepts. Consequently, in these countries costing of health care services is a routine in the hands of professional cost accountants. The literature on general cost accounting [13-19], costing of health services [20-24] and of hospital services [25-34] is enormous.

Since the end of the 1980s, a substantial number of papers have indicated the importance of costing health care services in developing countries [35-46], but our knowledge of provider costs in developing countries and in particular in least developed countries is still limited and the quality of studies is heterogeneous. Vaca, Kreider & Kreider were among the first to cost health care services in resource-poor countries. They conclude „that up-to-date and accurate book-keeping was not followed in many cases". Almost all lacked statistical information on the results of their projects. Among justifications for this were remarks like: ‚We're not working to fill out forms and show statistics.' [...] This would seem to be a common attitude among project staff. [...] Health workers aren't trained as economists and accountants and few projects have the money or inclination to employ such people themselves. It's little surprise that questions of financing sometimes never go beyond the short-term problem of obtaining funding and supplying satisfactory accounts to donors" [47].

This analysis was followed by a number of studies and reviews. In 1990, Mills assessed 30 studies of hospital costs in developing countries and notes that all of them were based on secondary data, i.e., existing accounts from the hospitals were used without consideration whether they were correct or complete [36,37]. The same approach was followed by the Christian Medical Commission (CMC, Geneva) which sent out questionnaires in order to assess the costs of hospitals. They had to realize that „there were wide variations in thoroughness in use of raw data, analysis and reporting" [48]. The quality of 27 studies on hospital costs analysed by Shepard, Hodgkin & Anthony [41] was higher, but only five of them were from least developed countries.

Meanwhile a number of studies invested effort to check and correct accounting data from health care institutions or programmes and search for missing figures. Our knowledge of costs of hospitals [49-51], districts [52-54] and specific diseases has grown. In particular, the costs of HIV/Aids care and prevention are thoroughly analysed [55-57]. However, the methodologies of these studies strongly differ and they all focus on small samples without being representative of the entire health care system of an entire nation.

One attempt to develop an insight into the costs of health care services in developing countries is the WHO-CHOICE project which has also contributed to our understanding of health care costs [34,58]. However, the 'Costit Model' of the WHO [59] is just a methodological frame where existing data from small-scale studies is entered. Currently, there is not a single study summarizing the cost of the entire health care system of an entire country on all levels of health care, in all regions and of all trustees. We know only very little about the costs of a health care system. In the case of Kenya we respond to this deficiency by presenting a costing model that provides reliable data for the entire health care system.

## Methods

The methodology section consists of four sub-sections. Firstly, we will describe the selection of the health care institutions that were part of this study. Secondly, we will explain the process of data collection in these institutions. Thirdly, the costing procedures will be presented. Fourthly, we will give basic information on the Kenyan Health Sector Costing Model Data Base containing the results of the first three steps.

### Selection of Health Care Institutions

The methods employed to obtain provider based costing data comprised facility surveys, face to face interviews and the review and analysis of secondary data sources. Based on the Kenya Health Care Facilities List of the Ministry of Health with 4002 facilities we stratified the health care institutions according to health care level (2: Dispensary; 3: Health Centre; 4: District Hospital; 5: Provincial Hospital; 6: Tertiary Hospital), trustee (Government, Faith Based/Nongovernmental Organisations, Private-for-Profit), capacity (e.g. number of beds) and location (urban vs. rural and different provinces). Some fields were empty, e.g. there is no tertiary hospital in many provinces, but 207 strata remained. If more than one institution was in a stratum, we took a random sample so that in total 207 health care facilities were included in the original sample. To our knowledge this is one of the highest sample sizes of any costing study conducted in developing countries, and this coupled with the depth of data collection, renders the Kenyan costing study the most comprehensive of its kind. After the survey, 53 health facilities had to be omitted from the analysis as their data sets were incomplete or unreliable. Table 1 exhibits the number of facilities in the analysis.

**Table 1:** Study facilities by level and trustee

	Government	Faith Based Organisation	Private-for-Profit	Total
Level 2 = dispensary	20	24	10	54
Level 3 = health centre	27			27
Level 4 = district hospital	20	10	11	41
Level 5 = provincial hospital	7			7
Level 6 = national hospital	2		1	3
Nursing Home		1	12	13
Administration (District and Provincial)	9			9

Total	85	35	34	154

At the targeted facilities, a total of 1369 patients were randomly selected and asked about their demand-sided costs.

To obtain information on super-overhead administrative function at central, provincial and district health level staff was purposefully selected using function (e.g. finance, head of administration or Human Resource) as the principle criterion.

### Data Collection

The collection of data followed the standards described in section 2. Based on two other studies of GIZ in Kenya (private-for-profit hospitals, faith-based hospitals) [60] and the excellent long-term relationship of the leadership of GIZ health sector programme in Kenya with the key-stakeholders of the Ministries, the faith-based health care providers and the private-for-profit providers (e.g. CONSORTIUM) our research teams did not experience any resistance.

In each health care facility, a questionnaire was used to obtain information on activities, expenditure, in-kind supplies received, staffing numbers as well as use of space and equipment. The questionnaire was modified for use in district and provincial offices to reflect a stronger emphasis on public health and administration rather than personal curative care. The questionnaire was pre-tested in 13 facilities covering at least one of each type of facility at all levels of service (level 2-6) and all trustees to examine its capability to capture essential information on costs and their distribution across the different cost centres at the facility level.

Data on recurrent costs were assembled in a variety of ways. The costs of medical and non-medical supplies, fuel, maintenance etc. were collected directly from facilities using a standard questionnaire through which inter-observer variability was minimised. Data collection was based on face-to-face interviews and access to records whilst at each facility.

All statements of interviewees were reconfirmed with institutional documentation in order to limit the recall-bias. These documents were either obligatory by law (e.g. budgets, budget comparison reports, income & expenditure accounts, balance sheets, HMIS statistics) or standards of good business practice, such as the pay-roll. Wherever possible, we relied on audited accounts (e.g. by the Treasury of Kenya). No use was made of postal or self-completed instruments. Data were collected for two financial years 2005/2006 and 2006/2007. These were averaged for the purposes of the model with the 2005/2006 figures increased by a standard rate of inflation (5%). All currency units were converted to Euro with an exchange rate of 90.66 Ksh/€. Both physical and financial resources utilised were documented. Where supplies were obtained in-kind their value was imputed from information on their value (mainly pharmaceuticals).

For a sub-sample of 70 institutions, an additional questionnaire was used to obtain the actual costs of treating patients for all 59 conditions included in the Kenya Essential Package of Health (KEPH). The questionnaire included a more detailed set of questions, such as medicines (by age group), staffing inputs, laboratory tests and examinations as well as the number of patients treated in an inpatient and outpatient setting in order to allow a detailed costing of each services package for the KEPH conditions.

In addition to the costs of the health care provider, we had to obtain information on the costs of the demand side. This became necessary as a major component of treatment costs is paid directly by the patients (or their relatives) without entering the books of the health care institutions. In particular drugs and medical supplies have to be bought outside of the health care facility in private pharmacies as the health care provider is frequently out of stock. Consequently, merely accounting of the expenditure of drugs and medical supplies of the health care providers would underestimate health care costs. In addition, it would also constitute a bias as public facilities suffer much more often from stock-outs than private health care institutions so that the costs of public health care.

Thus, standardized face-to-face exit interviews of both inpatients and outpatients were made to study the household contribution. In addition to the costs of drugs and medical materials purchased outside the health care provider, the questionnaire also asked for official and unofficial user fees and the costs of transport to and from the facility. These additional household costs were not utilized in this study as it focuses on the provider-side costs. However, they are of relevance for the Ministry of Medical Services and the Ministry of Public Health and Sanitation as they give some insight in the total cost-of-illness in Kenya.

We also calculated the expenditure of the Ministry of Health at central, provincial and district offices. For this purpose, a questionnaire and semi-structured interviews were employed.

Finally, an expert team of four (two clinicians, a laboratory expert and a pharmacist) was formed under the leadership of the Ministry of Health to develop standard treatment schemes for the KEPH conditions to permit calculation of normative as well as actual costs.

### Costing

**Figure 1:**
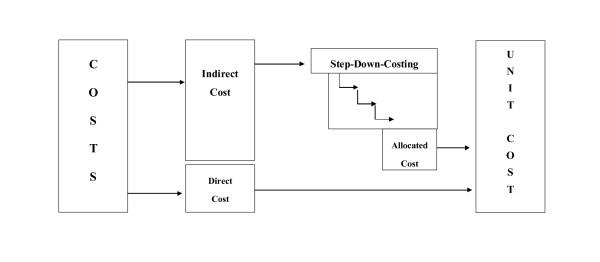
**Concept of Step Down Costing**.

Based on the questionnaires the total costs of each health care institution and of each condition of the Kenya Essential Package of Health (KEPH) were calculated. We applied the accounting standard of a step-down allocation as most studies referenced in section 2 (see Figure 1). This involves three steps. Firstly, costs of each department and of the entire hospital are collected. Costs which are induced by a single cost unit (e.g. outpatient visit, operation) are called direct cost and are allocated directly to the respective cost unit. Costs which cannot be allocated directly to a costing unit are called indirect costs and are allotted to the cost centre where they occur. Cost centres can be support centres (e.g. administration, laundry, kitchen) or final cost centres (e.g. outpatient department, paediatric ward, surgical ward).

Secondly, indirect costs are allocated step by step from the support centres to the final cost centres. The costs of the first service cost centre are allocated to the other cost centre and then to the final cost centre. Afterwards the accumulated costs of the next service cost centre are apportioned in the same way. This procedure is repeated step by step until all service cost centres are allocated to the final cost centres.

Thirdly, we calculate the allocated indirect cost per service unit in each final cost centre by dividing the total cost of each final cost centre by an appropriate measure of its output (e.g., number of outpatient visits, number of inpatient days). The total costs per service unit are then the sum of this term and the direct cost per service unit of the first step. For instance, the cost of an inpatient with pneumonia is calculated by adding the direct costs of the intravenous antibiotics to the product of cost per patient day in the TB-department and the lengths of stay.

In this study we used up to 70 cost centres (tertiary hospitals). The final cost centres were the outpatient department, the different wards of the inpatient departments and the outreach. Medical supplies, medicines and staff costs, which are directly associated with the treatment of specific KEPH conditions, were seen as direct cost.

A number of cost items require a more detailed discussion:

• Personnel: Staffing costs were based on the payroll of the respective health facility. For the public sector this was easily obtained from the Ministry of Health. For the private and NGO/FBO sector average incomes collected by the facility survey were used by default. Where this data were not available, the equivalent public sector salary is assumed. In some cases this may lead to a slight underestimate of the costs of FBO/private sector staffing although this error is not thought be large. Staffing costs include both the basic salary and allowances which in the public sector can account for 50% or more of staff remuneration. When costing the individual KEPH services the full salary plus allowances was used as the basis for valuing direct staff time. A cost per minute of time was computed as by dividing the base cost by the expected number of working minutes per year. The model base values assume that each member of staff works on average 220 days per year, equivalent to a five day working week plus holiday entitlement. It is assumed that each member of staff is available to provide direct services for 6 hours per day.

• Equipment and vehicles: We calculated the annual depreciation charge of equipment and vehicles based on a straight-line method. If no other information was available, we used an expected length of life of 10 years for general equipment (furniture etc.), 8 years for medical equipment and 8 years for vehicles. The estimate of the initial value was based price lists provided by the Ministry of Health along with information on the standard equipment that each level of facility should be equipped with. The researchers assumed that the prices and standards equally apply to NGO/FBO and private facilities. In the early stages of the study an attempt was made to cost the actual equipment used by facility type, but data collected proved too fragmentary to provide a consistent estimate across facilities. The only exception to this is level 6 facilities where no standards as regards equipment and prices exist. Here, the study estimated the actual costs of equipment at the facility level.

• Buildings: The annual depreciation charges of buildings were based on a straight-line method with an estimated length of life of buildings of 30 years. Based on the questionnaire, this statistic could be adjusted if the quality of buildings required it. The initial costs were calculated as the product of the building size [sqm] and the building cost per square metre. The latter was estimated by civil engineers and adjusted to the region.

• Administration: The costs obtained from the Ministry of Health and its decentralised functions were analysed and apportioned between its super-overhead administrative function and direct service provision.

• Demand-side costs: As described above, demand-sided costs were used to adjust the overall costs by level and function to take account of costs not recorded in facilities. For instance, many patients from government hospitals had to buy drugs from private pharmacies because the government facility was out of stock. We added these costs to the cost of the government hospital in order to determine the real resource consumption of treating a patient and not only the expenditure of this institution. Because of the imprecise diagnostic information obtained from the patient exit survey as well as the sample size it was not possible to break these data down by KEPH condition or even department. Demand-sided cost arising outside the facility could hence only be apportioned as an average to each outpatient and inpatient at each facility type.

• Normative cost: The normative cost of standard treatment guidelines for each KEPH activities was derived from the optimal medical supplies needed to treat the condition and staff time required for diagnosing, treating and nursing patients based on the recommendations of the expect committee under the wings of the Ministry of Health. This costing information is added to the fixed costs derived from the generalised costing to provide an estimate of the costs of each element of the KEPH.

The methodology applied in this study is a standard costing method and has been widely utilized in the research referenced in section 2. However, this study covers 207 institutions on five different levels of health care, about 70 cost centres and 59 costing units. This required a well-structured and professional approach to data collection, entry and presentation.

### Kenyan Health Sector Costing Model Data Base

All hand-written costing data was double-entered into Epi-Info and then uploaded to the Excel model to complete the step-down allocation process. All data is available in Software called "Kenyan Health Sector Costing Model". The model is operated by GIZ, and the data is utilized by the Ministry of Medical Services and the Ministry of Public Health and Sanitation (since the original Ministry of Health was recently split into two ministries).

The model produces the actual costs of the year of survey, the normative cost of the same year and prediction for actual and normative costs for future years. The model distinguishes between all levels of health care services and all trustees. A limited number of scientists with special training has access to the data base in order to simulate scenarios, such as changes in the work load, share of conditions (e.g. rate of Caesarean section), staffing levels, salaries, and prices in other input factors (e.g. drugs). Based on this data base we will present some basic results of actual costs of health care services in Kenya of the financial year 2006/2007.

## Results

In this section we present the total, unit costs and costs by diagnosis. We further analyse the cost-responsiveness behaviour of health care costs in Kenya.

### Total actual cost of health care services

Based on the model we can calculate the total cost of health care services in Kenya in the financial year 2006/2007 as 63 billion Ksh which is equivalent to 690 million Euros or 18.65 Euro per capita^1^. As table 2 shows, some 54% of this cost is incurred by public sector facilities, 17% by Faith Based (FBO) and other Nongovernmental (NGO) facilities and 23% in the private sector. Some 6% of the total cost is due to the overall administration provided directly by the Ministry and its decentralised organs. Table 2 also shows that around 37% of this cost is absorbed by salaries (52% of recurrent funding) and 22% by drugs and medical supplies (28% of recurrent). The percentage of staffing costs as a percentage of total facility cost varies across sectors and levels with more than 70% of cost in public outpatient facilities and less than 20% in those private facilities which are highly specialised on sophisticated equipment and well-off clients.

**Table 2:** Summary total costs of health services in Kenya [2006/7]

	Drugs and other supplies	Staffing	Other recurrent cost	Fixed cost	Total
Community (Public)	398.041€	233.770€	-€	91.992€	723.803€
Dispensary (Public)	12.927.460€	24.728.165€	1.813.250€	13.616.947€	53.085.822€
Health Centre (Public)	6.125.265€	20.227.027€	1.711.340€	9.587.565€	37.651.197€
District Hospital (Public)	42.745.105€	76.685.778€	13.503.152€	62.136.920€	195.070.955€
Provincial Hospital (Public)	5.434.795€	16.563.988€	2.361.242€	4.098.631€	28.458.656€
Tertiary Hospital (Public)	9.536.341€	28.296.419€	5.281.557€	4.251.671€	47.365.988€
Nursing Home/Enhanced HC(Public)	206.159€	456.053€	47.458€	142.743€	852.413€
Dispensary, Health Centre (FBO/NGO)	17.067.193€	10.931.167€	4.474.100€	13.846.061€	46.318.522€
District Hospital (FBO/NGO)	16.619.036€	19.528.286€	8.954.498€	25.072.792€	70.174.612€
Nursing Home/Enhanced HC (FBO/NGO)	45.077€	237.802€	45.778€	137.972€	466.629€
Dispensary, Health Centre (Private)	27.602.561€	32.701.781€	7.688.156€	34.615.153€	102.607.650€
District Hospital (Private)	8.098.325€	8.729.259€	3.797.198€	19.077.125€	39.701.906€
Tertiary Hospital (Private)	7.608.513€	3.203.292€	3.543.382€	4.251.671€	18.606.858€
Nursing Home/Enhanced HC	1.709.668€	1.460.479€	929.799€	781.843€	4.881.790€
District Administration	99.635€	8.629.723€	7.006.815€	-	15.736.173€
Provincial Administration	-	982.387€	610.011€	-	1.592.398€
Ministry of Health	-	6.138.911€	25.512.818€	-	31.651.729€

Total	156.223.174€	259.734.287€	87.280.555€	191.709.086€	694.947.102€

The ratio of primary care (Level 1 to 3) versus secondary care (Level 4 to 6) costs is 1:2. The results also reveal that 53% of the costs at service level are consumed by outpatient services and 47% by inpatient service provision. Unfortunately it was not possible to distinguish between dispensaries and health centres of Faith Based and other nongovernmental health institutions as their level of care was almost identical and the sample is not large enough to discriminate. The same applies for level 2 and level 3 facilities of private-for-profit organisations.

### Cost per Service Unit

**Figure 2:**
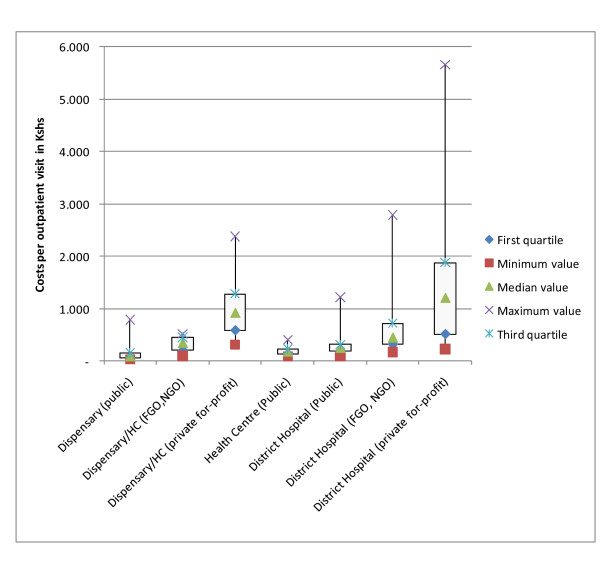
**Distribution of costs of outpatient visits**.

**Figure 3:**
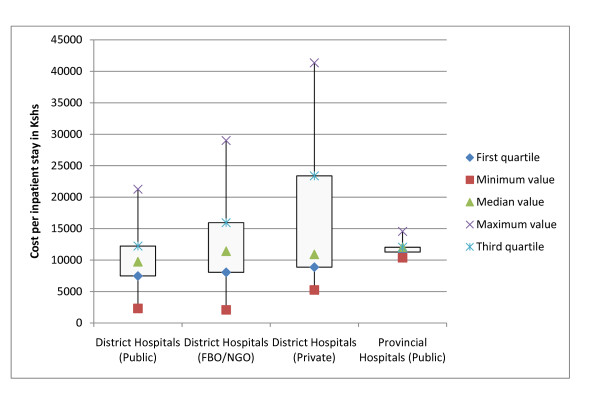
**Distribution of costs in inpatient services**.

Figure 2 compares the costs of outpatient visits at level 2 (Dispensaries), level 3 (Health Centres) and level 4 (District Hospital), Figure 3 exhibits the cost per inpatient stay in hospitals. Costs for outpatient visits and for inpatient stays vary substantially across the different types of providers. For public facilities the spread is limited when interpreted for each level of care, but private for-profit and not for-profit (NGO, FBO) facilities have a wide spread of cost per service unit. The median costs per outpatient visit are comparable for level 2 and 3 facilities between public and private not-for-profit facilities, but private for profit facilities tend to have a much higher median per level of service when compared to public facilities and private not-for-profit.

The costs of private-for-profit institutions differ strongly and in particular the costs of inpatient stays have a wide range. Some private-for-profit health care institutions have enormous unit costs, charge tremendous fees and serve the very rich. At the same time, some private-for-profit institutions offer low quality at low costs and do not serve the rich. This suggests that the average is not reflecting the reality of Kenyan health care costs.

The model calculates the cost per outpatient, the cost per admission, and the cost per bed-day. Table 3 exhibits the average unit cost by level and type of provider. The ratio between the cost per admission and the cost per bed-day is roughly the average length of stay. Again it was very difficult to distinguish dispensaries and health centres of Faith Based Organisations, other Nongovernmental Organisations and private-for-profit organisations. Some so-called "bedded dispensaries" are officially on level 2, but work as level 3 facilities, whereas some health centres of level 3 had no admissions and functioned as level 2 facilities.

**Table 3:** Average unit costs [Ksh]

	Cost per outpatient visit	Cost per admission	Cost per bedday	Ratios	
				Cost per admission/cost per bedday	Cost per bedday/cost per outpatient visit
Dispensary (Public)	174	-	-	-	-
Health Centre (Public)	223	3,500	3,500	1.0	15.7
District Hospital (Public)	518	12,970	2,186	5.9	4.2
Provincial Hospital (Public)	434	12,953	1,885	6.9	4.3
Tertiary Hospital (Public)	1,405	48,474	4,921	9.9	3.5
Dispensary, Health Centre (FBO/NGO)	633	2,242	4,194	0.5	6.6
District Hospital (FBO/NGO)	947	15,110	3,746	4.0	4.0
Dispensary, Health Centre (Private)	850	5,614	11,871	0.5	14.0
District Hospital (Private)	1,592	47,491	8,300	5.7	5.2
Tertiary Hospital (Private)	2,277	96,857	18,704	5.2	8.2

The ratio of the cost per bed-day and the cost per outpatient visit is useful to compare the workload of health care institutions. This ratio differs strongly, but it is higher than the rate of 1:2 frequently given in the literature (Table 3) [61]. This calls for further investigation. One reason might be the poor occupancy rate of the inpatient department in some health centres. Based on this data we can state that most health centres function as dispensaries with the costs of district hospitals.

As expected, the costs of primary care facilities per service unit are lower than the cost of district hospitals. However, the costs of public provincial hospitals are lower than of district hospitals. The immediate cause for this deviation from the trend is not known, and therefore warrants further investigation. One plausible reason is that higher specialisation and higher levels of bed occupancy (90% vs. 107%) leading to improved economies of scope and scale respectively.

**Figure 4:**
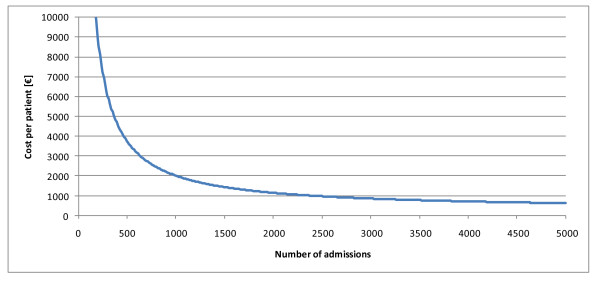
**Average cost per patient in private hospitals (district level) [€]**.

Based on the Kenyan Health Sector Costing Model and the strict distinction between variable and fixed costs we can calculate the unit costs at an occupancy rate of 85% (standard) (Table 4). The consequences are dramatic for private facilities. As Figure 4 shows, the cost per patient falls strongly with an increasing workload, i.e., after increasing the occupancy rate of private district hospitals to 85% the cost per admission are halved. This indicates a major problem of under-utilization of private-for-profit hospitals.

**Table 4:** Comparison of actual and standard unit costs in district (Level 4) hospitals [Ksh]

	Actual Cost		Standard Cost	
	Per admission	Per bedday	Per admission	Per bedday
Public	12,970	2,186	12,032	2,106
NGO/FBO	15,110	3,746	13,658	2,865
Private	47,491	8,300	23,340	4,520

### Costing Specific Diagnosis

As described in section 3, unit costs of KEPH conditions comprise two cost elements, direct and indirect costs. The direct component refers to the cost of medicines and supplies and key staff directly involved in the treatment of a KEPH condition. The indirect component is based on an apportionment of the overall costs of running the facility to individual KEPH services. To obtain the approximate cost of treating a KEPH condition the direct cost of an episode is added to the appropriate overhead. Table 5 shows results for three important diagnosis and the different health care institutions in Kenya.

**Table 5:** Average unit costs for specific KEPH treatment episodes [Ksh]

	Cost per normal delivery	Cost per malaria case (children < 5 years of age)	Cost per malaria case (children & adult ≥ 5 years of age)	Cost per case of diarrhoea (children < 5 years of age)
Dispensary (Public)	874	217	202	948
Health Centre (Public)	4,464	1,345	1,278	1,751
District Hospital (Public)	3,764	1,243	1,209	1,612
Provincial Hospital (Public)	3,349	1,139	1,124	1,556
Tertiary Hospital (Public)	8,900	3,355	3,177	4,362
Dispensary, Health Centre (FBO/NGO)	7,900	2,218	1,999	2,413
District Hospital (FBO/NGO)	6,281	1,035	1,944	2,355
Dispensary, Health Centre (Private)	10,976	3,175	2,795	3,449
District Hospital (Private)	13,828	3,905	3,435	4,097
Tertiary Hospital (Private)	21,414	6,350	5,507	6,689

Firstly, we find higher treatment costs per diagnosis in private facilities than in public facilities at the comparable level of care. This is in line with the results reported earlier.

Secondly, higher levels of care have higher costs per diagnosis. In particular dispensaries have much lower costs per diagnosis than any of the other facilities. This might be due to the fact that these institutions do not offer inpatient services and have lower overheads. At the same time, the severity of disease will be much lower in a dispensary than in the higher levels of care which require much more advanced technologies. It appears, however, that dispensaries are most efficient for milder diseases, i.e., it is worthwhile stressing the importance of the referral system of Kenya.

Thirdly, contrary to expectations the costs of treating any of the three selected conditions is higher in public health centres than in public district hospitals and higher still than in a provincial hospital. It is important to note that the direct costs tend to follow the anticipated pattern, i.e., higher levels of facilities have higher costs. Indirect costs of public health centres are, however, very high due to low occupancy. Consequently, the issue of public health centres has to be addressed in the health sector.

Fourthly, the costs per diagnosis of public provincial hospitals are lower than of public district hospitals. Again, the high occupancy of provincial hospitals results in low indirect costs per patient.

### Response Rates

Based on the questionnaires and the statistics of the health care institutions we could estimate the actual number of service units rendered and the theoretical demand. The latter was estimated by the Ministry of Health based on international standards [62-64] and the catchment population of the respective health care institutions. It was estimated that the actual demand for inpatient health care services in Kenya is only 17% of the theoretical need. For outpatient services it is 44%, so that on average 25% of health care needs are actually satisfied in professional health care services. However, it was estimated that there is a wide variety of coverage, e.g., for child delivery under skilled birth attendant it estimated by 37%, for malaria outpatient treatment by 72% and for septicemia only 5% [9].

**Figure 5:**
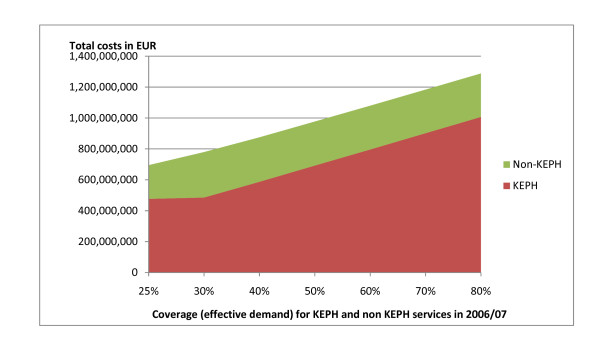
**Cost Responsiveness**.

The objectives of the health care reforms of Kenya can only be achieved if a higher share of the population is covered with health care until the entire population enjoys professional services. Consequently, we have to calculate the costs of the health care system as a function of the coverage. The development of the KEPH and Non-KEPH costs if the coverage increases from 25 to 80% (Figure 5). It is demonstrated that a minor increase (from 25 to 35% of coverage) will cause almost no effect on costs of KEPH conditions as the existing capacities are sufficient to cover this additional demand. For instance, it was estimated that the demand for health services would increase by approximately 40% (i.e. from 25 to 35% of theoretical needs) if Kenya made the political decision to remove user-fees for all KEPH services [65]. Figure 5 demonstrates that this will not have a major impact on the total costs of health care services.

Beyond that point additional demand will call for increased capacity. However, additional demand will not increase the health care costs proportionally as some costs are fixed. For instance, increasing the demand threefold (from 25% to 75%) will, for example, merely increase the costs by the factor 2.11 (Figure 5).

## Discussion

This paper provides an estimate of the total costs of health services in Kenya and its distribution across sector providers as well as levels of care. Compared with other studies from Sub-Saharan African countries (see State-of-the-Art) these costs are rather high, but Kenya has also higher gross national product per capita and higher salaries than neighbouring countries. For instance, in 2006 (mid-term of the study) the gross national product of Kenya was 527 US$ p.c. (not PPP-adjusted), whereas Tanzania (358 US$), Uganda (276) and Somalia (136 US$) had much lower GNPs p.c. Merely Sudan had a higher income per capita (643 U$), but this figure does not reflect Sudan's reality. It is skewed due to military aid and oil income. Generally, Kenya is the richest country in Eastern Africa, even if the average does not reflect regional and social disparities in this country (see Background).

The costing study reported in this paper was designed to inform policy makers about the total costs of health care services, where costs arise across the health sector and what the differences are in costs between the different types of providers and levels of care. Consequently, policy makers, politicians and donor agencies can utilize this data to base the Kenya health care reforms on costing evidence instead of guesses. Before the Kenyan Health Sector Costing Model existed only a very limited set of small-scale studies and budget reports existed which were neither representative nor reliable.

We suggest that the data presented in this study can be utilized in the Kenya Health Sector Reform process in the following areas:

• Budget impact: The actual costs of health care services in Kenya in 2006/07 were approximately 63 billion Ksh or 690 million Euros. However, with this amount we could cover merely some estimated 25% of the Kenya Essential Programme of Health for the entire population. Government decision-makers and donor agencies have to be aware of the fact that health care is expensive and that covering the entire population will be even more costly. However, investments in health care are usually regarded to be highly effective in a macro-economic perspective [66,67]. The Kenyan Government has strongly expressed its will to cover the entire population with health care services. Based on the assumption that 80% healthcare coverage is realistic, additional resources at the value of 431 million Euros would be required annually for services covered under the Kenyan Essential Package for Care (KEPH).

• Productivity: The figures indicate that Government facilities have generally lower costs per service unit than Faith Based Organisations, other Nongovernmental Organisations and private-for-profit organisations, i.e., without consideration of quality, Government facilities have a higher productivity than institutions of other trustees. This could have at least two reasons. Firstly, a poverty level of 46% [68] indicates that comparably cheap or free-of-charge services have a higher demand and therefore induce a higher utilization of services with correspondingly low costs per service unit. Indeed, a majority of the population seeks low-cost care due to financial constraints [69]. Secondly, the low unit costs of Government facilities might indicate a low quality of services. Indeed, literature and observations by the researchers reported widely on the frequency of drug stock-outs in public facilities in Kenya [68], an important indicator of quality. Higher utilization and lower quality of Government health care services can explain at least partly the comparably low unit costs of Government facilities.

The results of the Kenyan Health Sector Costing Model clearly indicate that the costs per service unit are generally lower at lower levels of the health care pyramid. Consequently, it is economically wise to strengthen the referral system, i.e., patient who can be treated in dispensaries should not be accepted by hospitals. There are many reasons why the referral system does not work, but based on this costing data the Kenya health care reform has to re-address the issue of self-referral.

• Spread of outpatient costs: The costs per service unit deviate strongly. For outpatient services the spread of costs is largest for private-for-profit facilities, signifying that either the productivity of privately run outpatient services is not homogeneous, or that quality varies widely within the sub-sector. This underlines the need to consider a variation in quality within the private subsector until further more conclusive studies are undertaken [70]. In Kenya it is definitely not true that the private-for-profit sector is only serving the rich with high-quality health care. Instead, the private-for-profit is also addressing the poorer strata of the society with affordable care (and most likely with a lower quality). This calls for a strengthening of all national efforts of quality assurance and regulation of the private sector to ensure consumer safety in the private health care sector. Thus, the Kenya health care reform has to stress the nationwide implementation of the Kenya Quality Model (KQM) in all health care facilities and the Ministry of Public Health and Sanitation as well as the Ministry of Medial Services have to accept their role as regulators of the health care market.

• Spread of inpatient costs: The average costs of public inpatient services are similar to those of private facilities. However, the range is quite high indicating that there are no homogenous inpatient services of private health care institutions in Kenya. Instead, the private health care sector is segmented into private hospitals for the richer strata of the society and private-for-profit hospitals for the poorer. In the political discussion in Kenya it is still assumed that private-for-profit hospitals are luxurious disease palaces for the super-rich. But this is not the case. There is a strong need to study the private health sector in more details. It is the impression of the researchers that we know by far too little about low-cost private health care services.

• Utilization: The generalised unit costs were found to be less in public facilities than in the private sub-sector, but the differences can be strongly reduced by increasing the utilization of private facilities. If, for instance, Kenya would cover 80% of the population with the Kenya Essential Package of Health (KEPH), we would either require a strong increase of the capacity of public facilities or utilize the private institutions to a much higher extent. As a majority of public facilities operates at full capacity and building new institutions is very expensive, efforts should be made to use the existing facilities and competences in the private sector. Not everybody in the Ministries of Health will readily accept the idea to utilize Faith Based and in particular private-for-profit organisations to a higher extent to cover the population with basic health care services. There is still an invisible rift between the public and the private sector. Some argue that private facilities are too expensive so that they are not suitable for the Kenya Essential Programme of Health. However, the health sector reform of Kenya focuses on Public-Private-Partnership and gives an explicit role to the private sector in providing health care services. Our study demonstrates that the cost per service unit (e.g. outpatient visit, hospital admission) of private-for-profit facilities are rather high in comparison to the institutions of other trustees. Our data also proves that the low utilization (e.g. number of outpatients, bed occupancy rate) of these private-for-profit institutions is a main reason for these high unit costs. Assuming normal price elasticity we can conclude, that the high unit costs in these institutions could be reduced if the Government of Kenya decided to pay for essential health care services irrespective of the owner of the health care institution.

• Staffing: The decreasing marginal unit cost with increasing utilization is based on the assumption that health care institutions could either meet the demand within their given labour capacity or acquire sufficient additional staff. However, hiring professional staff in rural health care institutions and in particular doctors for remote hospitals is quite difficult in Kenya. Professionals tend to work in cities (in particular Nairobi) and in high-level health care facilities. Our study results indicate that rural health care facilities are - on average - less staff intensive than urban facilities, and private-for-profit institutions attract more professional staff per service unit than government or faith-based/NGO institutions. The regulating bodies of Kenya must invest thought and effort to convince more professionals to work in rural places.

• Health insurance: The Vision 2030 and the Health Financing Strategy of Kenya have the objective to cover the entire population with essential health care services irrespective of an individual's income and wealth. The documents argue that this will in the long run be achieved by the introduction of a health insurance system. Some pilot projects are on the way (e.g. HAKI: Health for All Kenyans Through Innovations) to determine the prospects and rules of a possible health insurance system for Kenya. The Kenyan Health Sector Costing Model contributes to this development in several aspects. Firstly, it give a first insight into possible daily flat rate (e.g. for hospitals) and capitation (e.g. for dispensaries) as a starting point for the pilot districts of this new financing scheme. Secondly, it demonstrates that the same payments could be applied to health care institutions of all trustees if we control for quality. Insurance will reduce the individual financial burden and will allow patients to choose their provider so that we can anticipate that private health care institutions will attract more clients so that their unit costs will decrease. Consequently, this model calls for a rebate scheme where the same service (in quantity and quality) produces the same income for the provider irrespective of his ownership.

• Future KEPH: It is obvious that Kenya is in the epidemiological transition where chronic-degenerative diseases become more and more dominant. However, the current KEPH concentrates mainly on mother and child health care as well as infectious diseases. Therefore, KEPH will have to be adjusted regularly to allow for the new disease panorama of Kenya. The economic consequences are broadly unknown. The Kenyan Health Sector Costing Model gives at least some insights on the expected costs by showing reliable average cost per service unit (e.g. per patient day). This is a necessary - but not sufficient - condition of calculating the costs of new diagnoses to be included into KEPH. Other cost items, such as diagnosis-specific drugs, will have to be scrutinized additionally.

• Coverage: A coverage of some 25% of theoretical health care needs is quite dissatisfactory. Many patients do not seek professional health care due to long distances, high prices, poor quality and cultural reasons [64,71]. Our projections show that a mild increase of coverage has hardly any cost consequences as the direct costs of KEPH conditions are very low, and even strong increases in demand will not result in proportional growth of health care costs. Thus, health care reform must focus on instruments to reduce the barriers. Consequently, the Kenya Quality Model (KQM), the Health for All Kenyans through Innovations (HAKI) and the Mapping Study under the leadership of the Ministry of Medical Services and the Ministry of Public Health and Sanitation in cooperation with the German Development Cooperation (GIZ) are of high importance for an improved coverage. Their success will increase the health care expenditure in Kenya - but it is obvious that this increase will be moderate.

The Kenyan Health Sector Costing Model is based on a much higher sample of health care institutions than any of the studies references in section 3. It was scientifically supervised by international scholars and professionally implemented by the German Development Cooperation (GIZ) and the Ministry of Health of Kenya. Consequently, the quality of data is likely to be quite reliable in comparison with other studies on the costs of health care services in developing countries. However, the authors are aware of a number of shortcomings that limit the validity and representativeness of the data presented in this paper. Firstly, the model tried to cover also the cost of level 1 (community services). However, the wide diversity of community services, such as Aids-Control-Programmes, health education, nutrition programmes, gardening, road safety etc., made it very difficult to come up with reliable results. Secondly, we costed a large number of facilities in comparison to other studies. However, variability of costs, especially in the private sector may have warranted a larger sample in order to draw national policy conclusions from the study. Thirdly, facilities and patients were subjected to the costing exercise over a period of two months, which may under certain circumstances not be representative of the national average of costs, given that disease and consultation patterns are contingent on seasonal or other external variations. Finally, Nairobi based facilities were under-represented in the sample. Consequently, the results will correctly represent the situation in the rest of the country, but might under-estimate the total costs for the entire country as - at comparable levels of care - healthcare costs generated in Nairobi tend to be higher than healthcare costs generated in rural facilities.

## Conclusions

This paper presents the first publication of results of the Kenyan Health Sector Costing Model. The findings provide knowledge that health care costs in Kenya, efficiencies of health care provision and most likely quality are heterogeneous. This indicates that proposed health care reforms necessitate a flexible approach to account for the inherent differences in the health system. The study further demonstrated that significant room for improving the efficiencies of the public and private sector facilities exist. The public sector facilities are suggested to lack the necessary means to provide treatment according to national standards and protocols, whereby the availability of medicines appears to play a pivotal role in this. The private sector institutions are significantly more heterogeneous than the public sector with costs for services varying widely. It can only be hypothesised that this is mirrored in the variability of quality, but further studies need to be undertaken to validate this claim. What has been ascertained in this paper is that private facilities generate more costs for medicines and medical supply than public hospitals. Further we acknowledge that private facilities are frequently not used to their full capacity which has some relevant policy implications in view of Kenya's wish to meet Kenyan's needs for health services. At the time of the study the met need was estimated to be some 25% on average for all conditions. Besides the policy implications that the findings allude to, they also provoke economic considerations. Purchasers of health care services, i.e. the National Hospital Insurance Fund, private sector insurance companies or indeed the government can negotiate better prices if a steady and stable supply of patients to private health facilities can be assured. Measures to that end, such as capitation payments, are currently being explored in Kenya. Another measure, such as block contracting has so far not received attention at policy or political level. Idle capacity in the private sector can equally, but not exclusively be used for delivering maternal health services as a priority area, as the output-based aid voucher system operated by the Government of Kenya with support from the German Development Cooperation demonstrates.

The Kenyan Health Sector Costing Model is designed to base health care reform decisions on evidence. For this target, the German Development Cooperation (GIZ) invested some 500,000 € (full cost) to develop the model. It is agreed on that GIZ will also provide sufficient funds to update the database regularly so that Kenyan stakeholders are able to utilize these facts for evidence-based decision-making.

Meanwhile, the Ministries of Health of Kenya have started working with this tool, and key stakeholders in the areas of health finance are in the process of developing ways by which improved Hospital Management Information System (HMIS) data, routinely collected by health institutions, can be integrated into the database of the model. In addition, sample data from some pilot districts (e.g. from the pilots of the above mentioned HAKI project) is currently collected to up-date the data base. With this effort we can safeguard that fact-based efforts are employed to reach the target of the Vision 2030: a good health and reliable, equitable, affordable and sustainable health care services for the entire population of Kenya.

## Competing interests

The authors declare that they have no competing interests.

## Authors' contributions

SF and KH had the original idea of costing health care services in Kenya. TE was in charge of data collection and modeling. MM and SF drafted this paper. All authors read and approved the final manuscript.

## Note

^1 ^29.12.2006. Population: 37 million.

## Pre-publication history

The pre-publication history for this paper can be accessed here:

http://www.biomedcentral.com/1472-6963/11/128/prepub
